# Enhancement of Melanocortin-4 Receptor (MC4R) and Constancy of Kiss1 mRNAs Expression in the Hypothalamic Arcuate Nucleus in a Model of Polycystic Ovary Syndrome Rat

**DOI:** 10.22086/gmj.v0i0.1070

**Published:** 2018-05-29

**Authors:** Mohammad Hossein Nooranizadeh, Farhad Rahmanifar, Somayeh Ahmadloo, Zahra Shaaban, Mohammad Reza Jafarzadeh Shirazi, Amin Tamadon

**Affiliations:** ^1^Stem Cells Technology Research Center, Shiraz University of Medical Sciences, Shiraz, Iran; ^2^Department of Basic Sciences, School of Veterinary Medicine, Shiraz University, Shiraz, Iran; ^3^Department of Animal Sciences, College of Agriculture, Shiraz University, Shiraz, Iran

**Keywords:** Polycystic Ovary Syndrome, Melanocortin-4 Receptor, Kisspeptin, Light, Rats

## Abstract

**Background:**

Hypothalamic *Melanocortin-4* *Receptor* (*MC4R*) and kiss1/kisspeptin systems play roles in reproductive processes. This study was conducted to evaluate changes in *MC4R* and *kiss1* genes expression in the arcuate nucleus (ARC) of the hypothalamus and its relationship with polycystic ovary syndrome (PCOS) in rats.

**Materials and Methods:**

In the current experimental study, 24 female rats were randomly and equally allocated into nulliparous and primiparous groups and then were divided into two subgroups of PCOS and control. PCOS was induced by exposure to continuous light. Sex-related hormones were evaluated by radioimmunoassay or immunoradiometric assay. Expressions of *MC4R* and *kiss1* gene in the ARC of the hypothalamus of the rats were evaluated by real-time PCR. Histomorphometric alterations of ovaries were compared between groups.

**Results:**

Number of tertiary follicles and their size and number of atretic follicles in the PCOS subgroups were more than those in the controls (P<0.05) whereas the number of secondary follicles and corpus luteum in the PCOS subgroups were lower than those in the controls (P<0.05). Antrum and total diameters of tertiary follicles in the PCOS subgroups were greater and granulosa layer diameter was lower than those in the controls (P<0.05). The *MC4R* mRNA expression in the PCOS subgroups was 6.5-fold in nulliparous and 3.5-fold in primiparous groups more than their controls’ pairs (P<0.05). However, parity did not affect the expression of *MC4R* gene (P>0.05). The *kiss1* mRNA expression in the PCOS and control subgroups was not significantly different (P>0.05).

**Conclusion:**

Overexpression of *MC4R* gene after PCOS induction in the ARC of the hypothalamus may link to metabolic disorders of induced PCOS in the rats. However, alteration in the *kiss1* mRNA expression after PCOS induction was not observed in the rats.

## Introduction

Polycystic ovary syndrome (PCOS) is the most common metabolic and endocrine disorder in the reproductive-age women. PCOS causes infertility in women and is characterized by features such as clinical or biochemical hyperandrogenism, polycystic ovaries, and irregularity of menstruation [1]. Moreover, PCOS isassociated with obesity, insulin resistance, cardiovascular disease, and type 2 diabetes [2]. In this regard, free androgen levels in women with PCOS my correlate with obesity abnormalities [3].Additionally, the main reason for genetic obesity in human is mutation in *melanocortin-4 receptor*(MC4R) gene [4]. The MC4R is highly expressed in the hypothalamus. This gene is a G-protein coupled receptors that regulates eating behavior and body weight in humans and mice [5]. Moreover, the mutations and single nucleotide polymorphisms in theMC4R gene and other genes associated with obesity such as FTO demonstrated the development of obesity caused by PCOS [6, 7]. Therefore, evaluation of changes in gene expression of MC4R would be of great assistance to understand of its role in PCOS pathogenesis.Moreover, hormonal disorders occur during PCOS includinghypersecretion of luteinizing hormone (LH) and hyperandrogenism [8]. Hyperandrogenemia is dependent on insulin resistance and hypothalamic-pituitary-gonads (HPG) axis abnormalities including increased LH secretion [1]. PCOS can be due to alteration in feedback control of sex steroid hormones on gonadotropin-releasing hormone (GnRH) neurons [9]. In the hypothalamic arcuate nucleus (ARC), the kisspeptin/neurokinin B/dynorphin (KNDy) cells play an important role in rodents and humans by regulating GnRH neurons. In mammals, kisspeptin has a role in different reproductive and physiological conditions including spermatogenesis [10], pregnancy [11], milking [12, 13], estrus cycle [14, 15], malnutrition condition [16], and seasonal breeding [17]. Because they are important target tissues for sex steroids, the changes in KNDy peptides can be followed by neuroendocrine defects in PCOS [18]. There is evidence that kiss1 gene and the effects of GnRH/LH secretion by kisspeptin neurons may cause PCOS occurrence [3, 19]. Disturbances of HPG axis in PCOS, in part, may be due to alteration of expression of hypothalamic kiss1 mRNA that may involve in the pathogenesis of PCOS.

PCOS has been induced by the variety of methods in rodents [20]. One method to induce PCOS in rats is continuous lightening [21]. Continuous light for a long time creates similar characteristics of human PCOS in rats, including persistent estrus, anovulation, ovarian cysts, and increased levels of estrogen and androgen in plasma [21].Therefore, in order to remove the effect of exogenous sex hormones for induction of PCOS such as testosterone or estradiol on the expression of MC4R or kiss1 mRNAs, we selected constant light-induced PCOS model. Considering the probable roles of MC4R or kiss1miRNAs in the pathogenesis of PCOS, in the present study, we assessed their expressions in the ARC after induction of PCOS by constant light in rats.

## Material and methods

### Animals and PCOS Induction

All the procedures conducted in this experimental study were in accordance with instructions of Lab Animal Care Committees of Shiraz University of Medical Sciences and Shiraz University. In this study, 30 Sprague-Dawley female rats were purchased from and kept in the Center of Comparative and Experimental Medicine, Shiraz University of Medical Sciences. They included 12 nulliparous rats (38 days old and weight 177 ± 20 g), 12 primiparous rats (80 days old and weight 226 ± 20 g), and six nulliparous rats for ovariectomy procedure to usefor thereal time-PCR test as explained below. The nulliparous and primiparous rats were randomly allocated into two equal (n = 6) subgroups of PCOS and control. All rats of each subgroup were housed in one polycarbonate cage with free access to standard rat food and water at 22 ± 1 °C. Their body weights were weekly measured.

The rats in the PCOS subgroups were exposed to 24 h per day for 90 days continuous fluorescent light (intensity of 350 lux to 1 m2 on the floor) for induction of PCOS [21]. The control subgroups were housed in cages in 12 h/12 h light/dark photoperiod condition. In the PCOS and control subgroups, blood, brain, and ovaries were sampled after 90 days.

To use as a calibrator for real time-PCR, anesthetization of six nulliparous rats was performed by intraperitoneal injection of 100 mg/kg ketamine and 7 mg/kg xylazine (Woerden, Netherlands). Then, the rats were ovariectomized after a ventral midline incision. Brain sampling was performed after the two-week recovery period.

### Blood and Tissue Samplings

Blood samples were taken by cardiac puncture in the PCOS and control subgroups. Blood samples were stored in tubes without anticoagulant to clot. To separate serum, blood samples were centrifuged for 15 min with a speed of 2,000 rpm. Until the time of hormone assay, serum samples were frozenat -80°C.

After blood sampling, cervical dislocation was taken to remove the brain and isolating the ARC of the hypothalamus [22]. In brief, by an anterior coronal section, the diencephalon was dissected out. By a posterior coronal cut, optic chiasm and mammillary bodies were separated and, finally, the ARC was separated. Brain samples were stored in liquid nitrogen untilreal-time PCR analysis. Then, ovaries were removed through a ventral midline incision and were fixed in 10% formalin buffer.

### Hormone Assays

Serum testosterone concentrations were measured with 0.2 nmol/L sensitivity (catalog# RK-61M, Institut des Isotopes Ltd, Budapest, Hungary), serum estradiol concentrations with 2.7 pg/mL sensitivity (catalog# KIP0629, DIA Source Immuno Assays SA, Louvain-la-Neuve, Belgium), and serum progesteroneconcentrations with 0.05 ng/mL sensitivity (catalog# KIP1458, DIA Source Immuno Assays SA, Louvain-la-Neuve, Belgium) by radioimmunoassay (RIA) technique. Furthermore, using immunoradiometric assay (IRMA) technique, we evaluated serum follicle stimulating hormone (FSH) levels of with 0.09 mIU/mL sensitivity (catalog# RF01N, Gyeonggi-do, South Korea), serum LH levels with 0.22 mIU/mL sensitivity (catalog# RF03N, Gyeonggi-do, South Korea), and serum prolactin levelswith0.02ng/mL sensitivity (catalog# RF02NGyeonggi-do, South Korea).

### Histomorphometric Evaluation of Ovaries

Dehydration of the ovaries was donebygraded concentrations of ethanol and xylene. Then, they were embedded in paraffin wax. The ovaries were serially sectioned at thicknesses of 10 μm. Paraffin removal was done on one of every 10 serial sections in 60 °C. Then, selected sections were rehydrated in of xylene and decreasing serialconcentrations of ethanol. Finally, ovarian slices were stained with hematoxylin and eosin. To assess the type of follicles [23] and counting secondary, tertiary, and atretic follicles and corpora lutea, ovarian slices were observed with a light microscope (CX21, Olympus, Japan). Selected areas were photographed by a digital camera (AM423U Eyepiece Camera, Dino-Eye, Taiwan). Diameters of various structures including entire follicle, follicular antrum, granulosa and theca layers of secondary and tertiary follicles and corpus luteum were measured by Digimizer software (MedCalc Software bvba, Belgium)[21].

### Real-time PCR Analysis

Using liquid nitrogen, the isolated hypothalamic ARC samples (100 mg) were ground. Each sample powder in an RNase-free microtube was mixed in 1 mL Tripure Isolation Reagent RNX-Plus buffer. Then, total RNA was extracted with an RNX-plus Tripure Isolation Reagent (Roche Life Science, Branford, CT). The quantity and integrity of RNA were assessed by observing 18S and 28S rRNA bands on a 1% agarose gel. Nano-Drop ND 1000 spectrophotometer (Nano-Drop Technologies, Wilmington, DE, USA) was used to quantify the purified total RNA. To eliminate genomic contamination DNase, the test was performed with the DNase kit (Fermentas, St. Leon-Roth, Germany). The first strand cDNAswere synthesized in a 20 µL final volume from the DNase-treated RNA by the first strand cDNA Kit (Fermentas, St. Leon-Roth, Germany). Afterward, the *β-actin* was designed using Allele ID 7 software (Premier Biosoft International, Palo Alto, USA), primers target gene, MC4R,and kiss1, and reference gene (Table-1).

**Table-1:** Sequences of Real-Time PCR Primers and Amplification Reactions Conditions for Evaluation of the Relative Expression of Melanocortin-4 receptor (MC4R), Kiss1, and β-actin mRNA in the Rat Arcuate Nucleus of the Hypothalamus

**Primer**	**Sense and Anti-sense Sequence**	**Amplicon Length (bp)**	**Amplification Condition**
***MC4R***	GACGGAGGATGCTATGAG AGGTTCTTGTTCTTGGCTAT	116	15 min at 94℃, 40 cycles of 94℃ 10 s, 56.6℃ 15 s, and 72℃ 30 s
***Kiss1***	GCTGCTTCTCCTCTGTGT TAACGAGTTCCTGGGGTC	107	15 min at 94℃, 40 cycles of 94℃ 10 s, 58℃ 15 s, and 72℃ 30 s
***β*** ***-*** ***actin***	CCACACTTTCTACAATGAGC ATACAGGGACAACACAGC	169	15 min at 94℃, 40 cycles of 94℃ 15 s, 57.8℃ 20 s, and 72℃ 30 s

Real-time master mix (Yekta Tajhiz Azma, Tehran, Iran) was prepared in a 20-µL volume solution containing 1 µL cDNA, 4 ρmol of primer, and 1X SYBR Green buffer to perform relative real-time PCR. For quantitative measuring ofreal-time PCR data, the threshold cycle (CT) method was used to calculate the relative expression of the MC4R and kiss1 mRNAs. In details, using StepOne real-time PCR software (Applied Biosystems, Foster City, CA, USA), the CT for each sample was calculated. The amplification reactions are shown in Table-1.

Calculation ofrelative expressions of MC4R and kiss1 mRNAs to the expression of reference gene was done by the 2^-ΔΔCT^ equation [24], where ΔCT was the subtraction of the internal control CT value from the specific CT of the target gene.

The ΔΔCT was the subtraction of the ΔCT of each sample from an average of the ovariectomized calibrator rats.

### Statistical Analysis

All data from body weight, real-time PCR of the genes, hormone analysis, and histomorphometricanalysis were subjected to the normality evaluation byKolmogorov-Smirnov test.The normal data or log-transformed normalized data were analyzed by one-way ANOVA and LSD *post hoc* test (IBM SPSS Statistics for Windows, Version 22.0, Released 2013. Armonk, NY: IBM Corp).Means and standard error of grouped data were presentedin the graphs (GraphPad Prism version 5.01 for Windows, GraphPad Software Inc., San Diego, CA, USA).

## Results

### Body Weight Changesin the PCOS Subgroups

**Figure-1:**
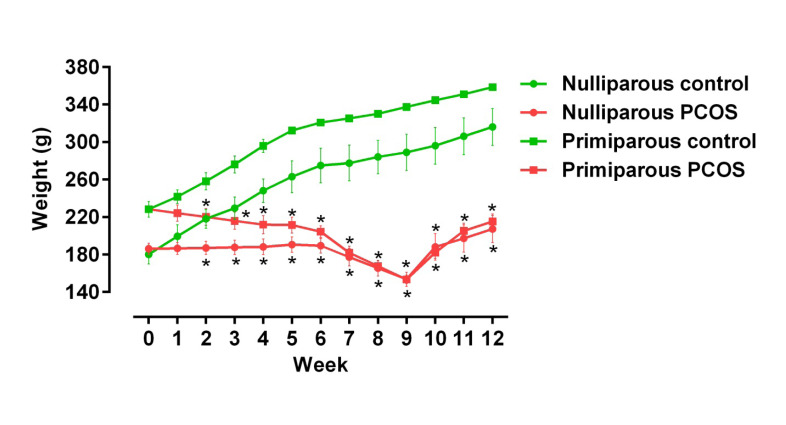
Evaluation of mean and standard error of body weight in primiparous and nulliparous polycystic ovary syndrome (PCOS) rats that were induced by exposure to constant light. Stars show statistically significant differences between control and PCOS groups (P<0.05).

In the nulliparous and multiparous rats, body weight in the PCOS subgroupdecreased until week 9 and then increased until the end of study (P<0.05, Figure-1). These alterations were significant between the control and PCOS subgroups in the both parities (P<0.05).

### Hormone Alterations in the PCOS Subgroups

**Figure-2:**
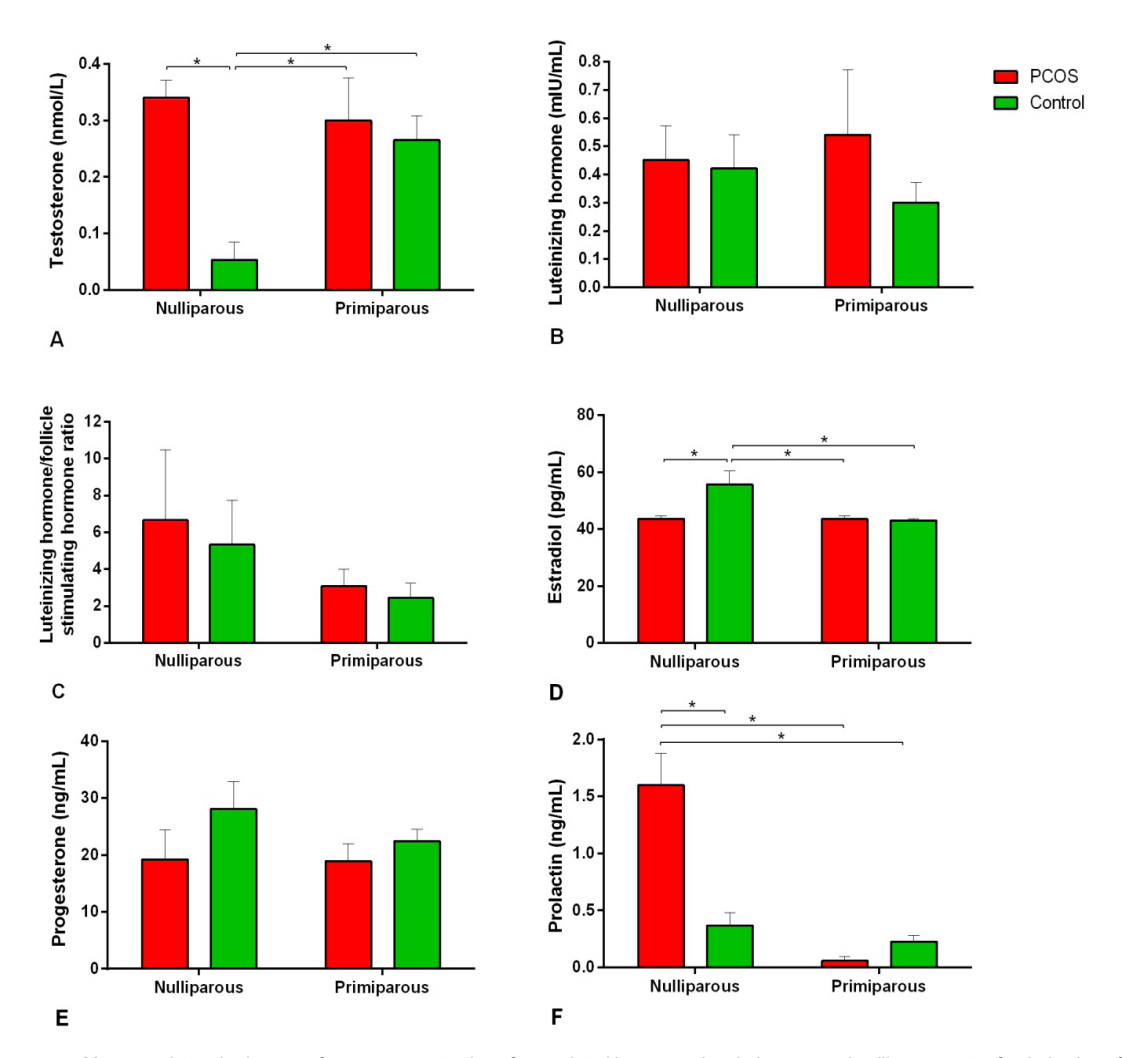
Means and standard errors of serum concentration of sex-related hormones in primiparous and nulliparous rats after induction of polycystic ovary syndrome (PCOS) by exposure to constant light: **A)** testosterone concentrations; **B)** luteinizing hormone concentrations; **C)** ratio of luteinizing hormone/follicle stimulating hormone concentrations; **D)** estradiol concentrations; **E)** progesterone concentrations; and F) prolactin concentrations. Stars show statistically significant differences between groups (P<0.05).

In the nulliparous rats, serum testosterone and prolactin concentrations in the PCOS subgroup were more than those in the control (P<0.001, Figure-2). In comparison, concentrations of estradiol in the nulliparous control were more than their counterparts in the PCOS subgroup (P=0.005).

Serum progesterone and LH levels and LH/FSH ratio were not different between the control and PCOS subgroups (P>0.05).

### Histologic and HistomorphometricChangesin the PCOS Subgroups

**Figure-3:**
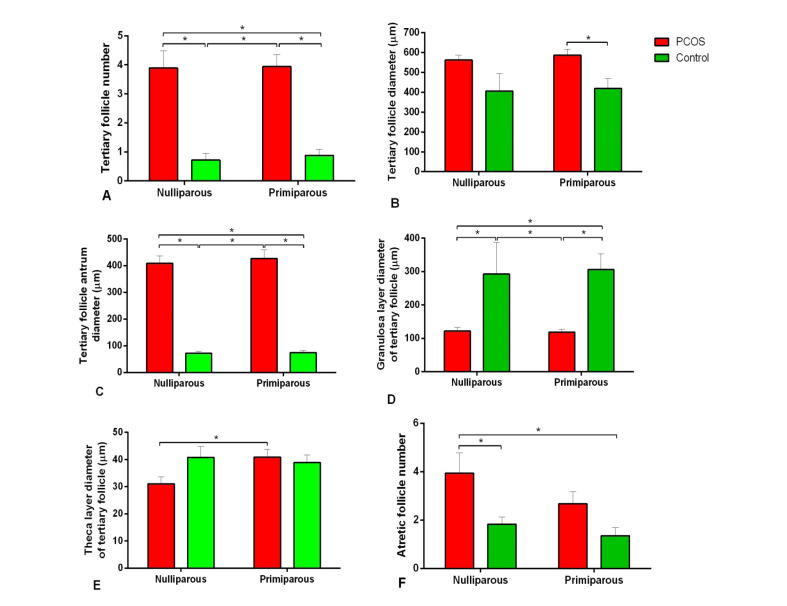
Means and standard errors of histomorphometric indices oftertiary follicles in primiparous and nulliparous rats after induction of polycystic ovary syndrome (PCOS) by exposure to constant light: **A)** tertiary follicle number;** B)** tertiary follicle diameters; **C)** tertiary follicle antrum diameters; **D)** granulosa layer diameter of tertiary follicle; **E)** theca layer diameter of tertiary follicle; and **F)** atretic follicle number. Stars show statistically significant differences between groups (P<0.05).

**Figure-4:**
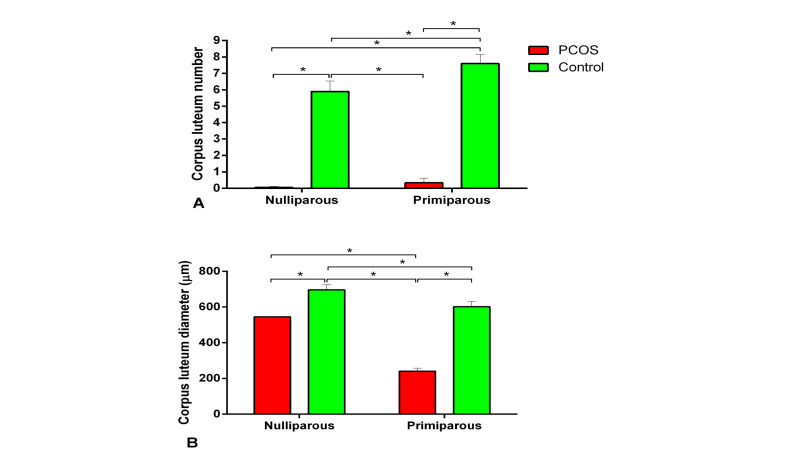
Means and standard errors of histomorphometric indices of corpus luteum in primiparous and nulliparous rats after induction of polycystic ovary syndrome (PCOS) by exposure to constant light: **A)** corpus luteum number; **B)** corpus luteum diameter. Stars show statistically significant differences between groups (P<0.05).

**Figure-5:**
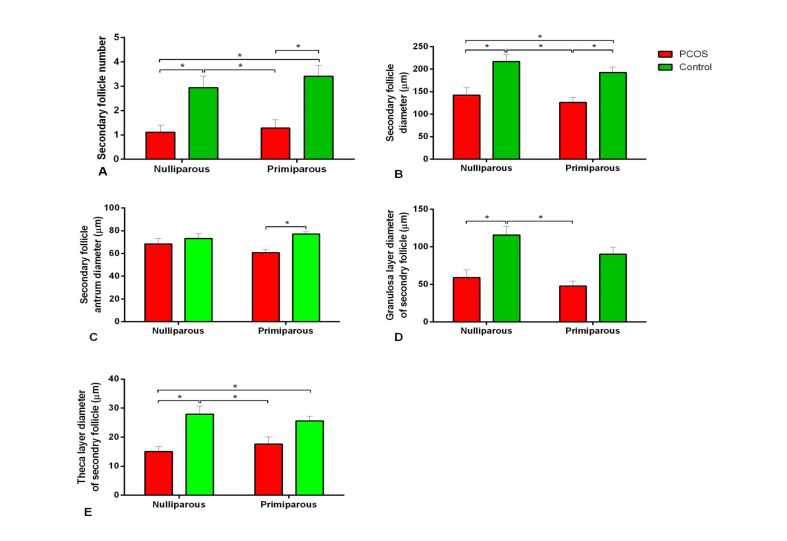
Means and standard errors of histomorphometric indices of secondary follicles in primiparous and nulliparous rats after induction of polycystic ovary syndrome (PCOS) by exposure to constant light: A) secondary follicle number; B) secondary follicle diameters; C) secondary follicle antrum diameters; D) granulosa layer diameter of secondary follicle; and E) theca layer diameter of secondary follicle. Stars show statistically significant differences between groups (P<0.05).

Histological comparison of the results between the control and PCOS subgroups showed that the number of tertiary or antral follicles and diameter of antral follicles in the PCOS subgroups were more than those of the control (P<0.05, Figure-3). In addition, atretic follicles of the nulliparous PCOS rats were significantly more than the control (Figure-3). Corpus luteum was not observedin the ovary of the PCOS subgroups (Figure-4). As shown in the Figure-5, the number of secondary follicles and their diameter in the control subgroupswere higher than those of the PCOS subgroups (P<0.05). Diameters of granulosa and theca layers of secondary follicles in the nulliparous control group were higher compared to their PCOS counterpart (P<0.05, Figure-5). In addition, the diameter of granulosa layers of tertiary follicles was significantly reduced in the PCOS subgroups (Figure-5). These follicular structure alterations by PCOS induction were more severe in the nulliparous rats than the primiparous ones.

### MC4R and Kiss1 mRNA Alterationsin the PCOS Subgroups

**Figure-6:**
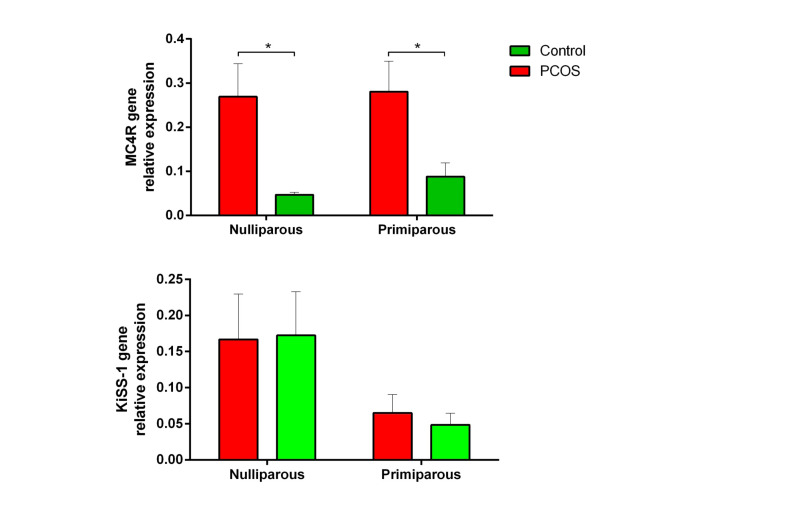
Means and standard errors of expression of MC4R and kiss1 mRNAs in the hypothalamic arcuate nucleus of primiparous and nulliparous rats after induction of polycystic ovary syndrome (PCOS) by exposure to constant light. Stars show statistically significant differences between groups (P<0.05).

The graphs in the Figure-6 showed that PCOS induction in rats by constant light exposure resulted in overexpression of *MC4*R mRNA in the ARC in both groups of nulliparous and primiparous (P<0.05). However, no change was observed in the expression of *kiss1* mRNA in the hypothalamic ARC of rats after induction of PCOS by constant light (P>0.05).

## Discussion

Induction of PCOS by continuous light in rats significantly increased expression of *MC4R*in the ARC of the hypothalamus. Consistent with our findings, expression of hypothalamic *MC4R* was increased in hyperandrogenic PCOS rats [25].

In addition, body weight increased after week 9; however, weight loss was observed in both PCOS groups before week 9. In agreement with our observations, associations between *MC4R* mutations, PCOS, and body mass index in human were shown in [26, 7]. MC4R playsa critical role in the regulation of energy homeostasis and body weight by controlling appetite and energy expenditure [27].

So, probable weight alterations in the PCOS rats are linked tochanges in gene expression of MC4R according to our findings and/or mutations of *MC4R* [26].

Although these factors are not evaluated in this study, it is shown that hypertension [25] and cardiovascular disease [28], which are other metabolic disorders related to PCOS, have an association with high expression of MC4R in the other rat models of PCOS.

Dysfunction of HPG axis and impaired of GnRH/LH pathway were observed in PCOS as a result of hypersecretion of LH and an increase in LH secretion is the most important feature of PCOS in women [1]. Some controversy findings show that dysregulation of GnRH neurons may be caused by a change in expression of kiss1/kisspeptin neuronal system involved in the pathogenesis of PCOS (Table-2). In our study, the constant light did not change *kiss1* gene expression in the hypothalamic ARC. These different findings may be affected by the model of PCOS induction. Availability of a suitable model to investigate the etiology and pathophysiology of PCOS has led to the creation of a variety of different models for induction of PCOS. The kiss1/kisspeptin hypothalamic system may besensitive to the regulating effects of exogenous sex steroids. Estrogens, progesterone, and even androgen have feedback effects on GnRH release via modulation of kisspeptin neurons [19]. In general, the role of abnormal expression of *kiss1* mRNA is not well established in the etiology of PCOS; thus, clarifying the role of the kisspeptin needs further investigation.

**Table-2:** Effect of Different PCOS Models in Rat that Were Induced by Exogenous Sex Hormones Manipulation on the Hypothalamic Expression of Kisspeptin Gene or peptide

**Method of Induction**	**Age of Induction**	**Duration of Induction**	**Method of Evaluation**	**Kisspeptin Expression**	**References**
**Dihydrotestosterone**	Postnatal day 18	26 days 60 days	Real-time PCR/ Immunohistochemistry	Decrease No effect	[33]
**Dihydrotestosterone**	Postnatal day 21	14 days	Real-time PCR	No effect	[34]
**Testosterone propionate / estradiol benzoate**	Postnatal day 0-3	90 days	Real-time PCR	Decrease	[34]
**Anti-progestin RU486**	Adult, 49 days	14 days	Immunohistochemistry	Increase	[35]

In our study, ovarian histological evaluation showed that the number of antral and atretic follicles increased in the PCOS group. An increase in the number of atretic follicles in the ovaries of rats after 100 days of continuous exposure to light has been previously observed [29]. After 13 weeks of continuous light, large cystic follicles in the ovaries along with thin granulosa cells were reported for Wistar rats [30]. Also, in the current study, corpus luteum was not observed in the PCOS group of the ovary, suggesting that ovulation is not carried out under continuous light condition. In another study, large antral follicles and absence of the corpus luteum in the ovary were observed after 91 days of continuous exposure[31]. Reduced number of secondary follicles in the ovary of PCOS rats was observed in our findings. Consistent with our findings, continuous light reduced growing follicles and elevatedatretic follicles in the same model of PCOS [31]. In general, alteration in the population of ovarian follicles indicated that continuous light for 90 days [21] can create typical features of PCOS in rats.

The hormonal evaluation showed an increase in the concentration of serum prolactin in the uniparous PCOS group. To sum up, high prolactin level was observed in PCOS patients [32].

Furthermore, testosterone levels increased in PCOS group compared to the control group. In line with our data, the same alteration in circulating testosterone has been reported [21]. In contrast with our findings, elevated testosterone levels were not observed in the same animal model [20]. In our study, serum gonadotropin levels (FSH and LH) did not change after PCOS induction. In the PCOS model with stable light, although changes in the LH levels were not observed, the FSH was decreased [20]. In the present study, serum progesterone levels did not differ between the control and PCOS groups, but estradiol levels were higher in the control group. Increased levels of estradiol and reduced progesterone levels were observed in a PCOS model of continuous light [20]. Therefore, continuous light can cause irregularities in sex steroid hormones in rats. However, common PCOS hormonal profiles in PCOS women could not be established in this model. Taken together, although continuous light caused ovarian cysts formation without exogenous hormonal manipulation of the hypothalamus; it could not completely make a similar hormonal profile to PCOS women in the rat model.

## Conclusion

Expression of *MC4R* gene in the ARC of the hypothalamus and increased body weight had a relationship with PCOS induction inrats. Therefore, changes in expression of *MC4R* gene may have a role in etiology of PCOS. However, no effect on expression of *kiss1* mRNA was observed in the hypothalamus after PCOS induction. Since changes were not observed in the serum LH levels, it can be justified by the lack of change in *kiss1* mRNA expression.

## Conflict of Interests

None of the authors has any potential conflict of interests associated with this research.
